# Tetra­butyl­ammonium *N*-benzoyl-6-nitro-1,3-benzothia­zol-2-aminide

**DOI:** 10.1107/S1600536810026504

**Published:** 2010-07-10

**Authors:** Quynh Pham Bao Nguyen, Sung Ok Kang, Taek Hyeon Kim

**Affiliations:** a Department of Applied Chemistry, Chonnam National University, Gwangju 500-757, Republic of Korea

## Abstract

In the title salt, C_16_H_36_N^+^·C_14_H_8_N_3_O_3_S^−^, the torsion angles within the cation reveal that one butyl group displays an *anti* conformation and the other three butyl groups show *gauche* conformations. The anion is almost planar, with a largest deviation of 0.166 (6) Å from the least-squares plane (r.m.s. deviation of fitted atoms = 0.052 Å). In the crystal structure, the component ions inter­act by means of weak inter­molecular C—H⋯O hydrogen bonds.

## Related literature

For the development of colorimetric chemosensors, see: Coll *et al.* (2007 ▶); Evans *et al.* (2006 ▶). For similar deprotonation reactions, see: Kang *et al.* (2009 ▶).
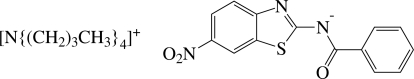


## Experimental

### 

#### Crystal data

C_16_H_36_N^+^·C_14_H_8_N_3_O_3_S^−^
*M* *_r_* = 540.75
Monoclinic, 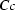

*a* = 7.9234 (7) Å
*b* = 25.059 (2) Å
*c* = 15.4916 (14) Å
β = 97.699 (2)°
*V* = 3048.2 (5) Å^3^
*Z* = 4
Mo *K*α radiation
μ = 0.14 mm^−1^
*T* = 223 K
0.22 × 0.11 × 0.10 mm

#### Data collection

Bruker SMART 1000 CCD diffractometer
Absorption correction: multi-scan (*SADABS*; Bruker, 2000 ▶) *T*_min_ = 0.979, *T*_max_ = 0.988
11371 measured reflections
5730 independent reflections
2547 reflections with *I* > 2σ(*I*)
*R*_int_ = 0.076

#### Refinement

*R*[*F* ^2^ > 2σ(*F* ^2^)] = 0.069
*wR*(*F* ^2^) = 0.169
*S* = 0.95
5730 reflections
347 parameters
2 restraints
H-atom parameters constrained
Δρ_max_ = 0.25 e Å^−3^
Δρ_min_ = −0.19 e Å^−3^
Absolute structure: Flack (1983 ▶), 1908 Friedel pairs
Flack parameter: 0.08 (12)

### 

Data collection: *SMART* (Bruker, 2000 ▶); cell refinement: *SAINT* (Bruker, 2000 ▶); data reduction: *SAINT*; program(s) used to solve structure: *SHELXS97* (Sheldrick, 2008 ▶); program(s) used to refine structure: *SHELXL97* (Sheldrick, 2008 ▶); molecular graphics: *ORTEP-3* (Farrugia, 1997 ▶) and *PLATON* (Spek, 2009 ▶); software used to prepare material for publication: *SHELXL97*.

## Supplementary Material

Crystal structure: contains datablocks global, I. DOI: 10.1107/S1600536810026504/lx2157sup1.cif

Structure factors: contains datablocks I. DOI: 10.1107/S1600536810026504/lx2157Isup2.hkl

Additional supplementary materials:  crystallographic information; 3D view; checkCIF report

## Figures and Tables

**Table 1:** Hydrogen-bond geometry (Å, °)

*D*—H⋯*A*	*D*—H	H⋯*A*	*D*⋯*A*	*D*—H⋯*A*
C28—H28*A*⋯O1^i^	0.98	2.24	3.166 (8)	157
C29—H29*A*⋯O2^ii^	0.98	2.39	3.353 (9)	166

Symmetry codes: (i) 
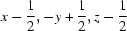
; (ii) 
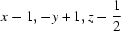
.

**Fig. 1.**
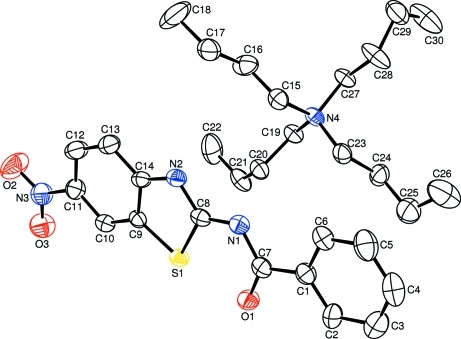
The structure of the title salt, with displacement ellipsoids drawn at the 50% probability level. H atoms are omitted.

**Fig. 2.**
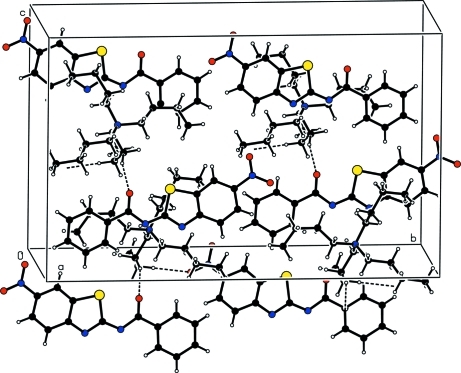
View of the unit-cell contents of the title salt. Hydrogen-bond interactions are drawn with dashed lines.

## supplementary crystallographic information

### Comment

The development of colorimetric chemosensors is attractive in supramolecular chemistry because they give a direct signal which is easily observed by the naked eye (Coll *et al.*, 2007). Most chemosensors contain –NH fragments which act as hydrogen bond donors for the binding of anions. However, the chromophore fragments for colorimetric signal may contain electron-withdrawing groups that enhance the acidity of the –NH protons of the binding site. Therefore, these acidic protons can be abstracted by the basic anions in the deprotonation process (Evans *et al.*, 2006). In some cases, it is difficult to establish a clear difference between a hydrogen bond donor binding process and a deprotonation process. The title salt was prepared on the study of the nature of interaction between *N*-(6-nitrobenzo[*d*]thiazol-2-yl)benzamide and tetrabutylammonium acetate (Kang *et al.*, 2009).

The asymmetric unit of the title salt, C_16_H_36_N^+^.C_14_H_8_N_3_O_3_S^-^, contains a tetrabutylammonium cation and a deprotonated anion of *N*-(6-nitrobenzo[*d*]thiazol-2-yl)benzamide (Fig. 1). The C15—C16—C17—C18 torsion angle of -179.9 (7)° displays the anti conformation for the four atoms of a butyl group within the cation, whereas the other three butyl groups show the *gauche* conformations with the torsion angles of -74.8 (7)° (C19—C20—C21—C22), -75.7 (8)° (C23—C24—C25—C26) and -65.9 (9)° (C27—C28—C29—C30)°. The anion is almost planar with the largest deviation of 0.166 (6) Å (O2) from the least-squares plane (r.m.s. deviation of fitted atoms: 0.052 Å). In the crystal structure, the component ions interact by means of weak intermolecular C—H···O hydrogen bonds (Table 1 and Fig. 2).

### Experimental

A solution of 6-nitrobenzo[*d*]thiazol-2-amine (0.300 mg, 1.54 mmol) and benzoyl chloride (0.148 ml, 1.28 mmol) in pyridine was refluxed for 24 h under argon atmosphere. Upon completion of the reaction, the reaction mixture was cooled to 0 °C, and poured into water. The formed precipitate was separated by filtration and washed with methanol, dichloromethane and ether and dried, to give a yellow solid *N*-(6-nitrobenzo[*d*]thiazol-2-yl)benzamide (0.192 g, yield 50%). Crystals of the title compound suitable for X-ray analysis were obtained in a deprotonation reaction that involved slow evaporation of an acetonitrile solution of *N*-(6-nitrobenzo[*d*]thiazol-2-yl)benzamide in the presence of excess tetrabutylammonium acetate at room temperature.

### Refinement

H atoms were positioned geometrically and allowed to ride on their respective parent atoms [C—H = 0.94 (CH), 0.98 (CH_2_) or 0.97 Å (CH_3_) and *U*_iso_(H) = 1.2*U*_eq_ or 1.5*U*_eq_(methyl C)].

### Crystal data

**Table:**

C_16_H_36_N^+^·C_14_H_8_N_3_O_3_S^−^	*F*(000) = 1168
*M**_r_* = 540.75	*D*_x_ = 1.178 Mg m^−^^3^
Monoclinic, *C**c*	Mo *K*α radiation, λ = 0.71073 Å
Hall symbol: C -2yc	Cell parameters from 1597 reflections
*a* = 7.9234 (7) Å	θ = 2.7–19.4°
*b* = 25.059 (2) Å	µ = 0.14 mm^−^^1^
*c* = 15.4916 (14) Å	*T* = 223 K
β = 97.699 (2)°	Block, yellow
*V* = 3048.2 (5) Å^3^	0.22 × 0.11 × 0.10 mm
*Z* = 4	

### Data collection

**Table:**

Bruker SMART 1000 CCD diffractometer	5730 independent reflections
Radiation source: fine-focus sealed tube	2547 reflections with *I* > 2σ(*I*)
graphite	*R*_int_ = 0.076
φ and ω scans	θ_max_ = 28.3°, θ_min_ = 2.1°
Absorption correction: multi-scan (*SADABS*; Bruker, 2000)	*h* = −9→10
*T*_min_ = 0.979, *T*_max_ = 0.988	*k* = −33→29
11371 measured reflections	*l* = −19→20

### Refinement

**Table:**

Refinement on *F*^2^	Secondary atom site location: difference Fourier map
Least-squares matrix: full	Hydrogen site location: inferred from neighbouring sites
*R*[*F*^2^ > 2σ(*F*^2^)] = 0.069	H-atom parameters constrained
*wR*(*F*^2^) = 0.169	*w* = 1/[σ^2^(*F*_o_^2^) + (0.0566*P*)^2^] where *P* = (*F*_o_^2^ + 2*F*_c_^2^)/3
*S* = 0.95	(Δ/σ)_max_ < 0.001
5730 reflections	Δρ_max_ = 0.25 e Å^−^^3^
347 parameters	Δρ_min_ = −0.19 e Å^−^^3^
2 restraints	Absolute structure: Flack (1983), 1908 Friedel pairs
Primary atom site location: structure-invariant direct methods	Flack parameter: 0.08 (12)

### Special details

**Table:**

Geometry. All e.s.d.'s (except the e.s.d. in the dihedral angle between two l.s. planes) are estimated using the full covariance matrix. The cell e.s.d.'s are taken into account individually in the estimation of e.s.d.'s in distances, angles and torsion angles; correlations between e.s.d.'s in cell parameters are only used when they are defined by crystal symmetry. An approximate (isotropic) treatment of cell e.s.d.'s is used for estimating e.s.d.'s involving l.s. planes.
Refinement. Refinement of *F*^2^ against ALL reflections. The weighted *R*-factor *wR* and goodness of fit *S* are based on *F*^2^, conventional *R*-factors *R* are based on *F*, with *F* set to zero for negative *F*^2^. The threshold expression of *F*^2^ > σ(*F*^2^) is used only for calculating *R*-factors(gt) *etc*. and is not relevant to the choice of reflections for refinement. *R*-factors based on *F*^2^ are statistically about twice as large as those based on *F*, and *R*- factors based on ALL data will be even larger.

### Fractional atomic coordinates and isotropic or equivalent isotropic displacement parameters (Å^2^)

**Table:**

	*x*	*y*	*z*	*U*_iso_*/*U*_eq_	
S1	0.70508 (16)	0.32664 (5)	0.33449 (9)	0.0502 (4)	
O1	0.6825 (5)	0.22458 (14)	0.3155 (3)	0.0623 (11)	
O2	0.8146 (9)	0.57535 (19)	0.3706 (4)	0.131 (2)	
O3	0.8142 (7)	0.52224 (19)	0.4779 (4)	0.0984 (16)	
N1	0.6279 (5)	0.26786 (17)	0.1823 (3)	0.0505 (11)	
N2	0.6516 (5)	0.36006 (18)	0.1718 (3)	0.0521 (11)	
N3	0.7979 (7)	0.5308 (2)	0.3993 (5)	0.0829 (16)	
C1	0.6094 (7)	0.1727 (2)	0.1884 (4)	0.0573 (15)	
C2	0.6156 (8)	0.1257 (2)	0.2357 (5)	0.0767 (19)	
H2	0.6405	0.1272	0.2967	0.092*	
C3	0.5860 (10)	0.0768 (3)	0.1957 (6)	0.102 (3)	
H3	0.5866	0.0454	0.2290	0.123*	
C4	0.5553 (9)	0.0746 (3)	0.1050 (7)	0.099 (3)	
H4	0.5379	0.0415	0.0767	0.119*	
C5	0.5504 (9)	0.1211 (3)	0.0569 (5)	0.088 (2)	
H5	0.5319	0.1197	−0.0043	0.106*	
C6	0.5723 (7)	0.1689 (2)	0.0985 (4)	0.0640 (16)	
H6	0.5621	0.2004	0.0652	0.077*	
C7	0.6423 (6)	0.2240 (2)	0.2350 (4)	0.0521 (14)	
C8	0.6573 (6)	0.3158 (2)	0.2190 (3)	0.0472 (14)	
C9	0.7132 (6)	0.3942 (2)	0.3138 (3)	0.0457 (13)	
C10	0.7471 (7)	0.4359 (2)	0.3727 (4)	0.0544 (14)	
H10	0.7650	0.4295	0.4330	0.065*	
C11	0.7536 (7)	0.4870 (2)	0.3401 (4)	0.0628 (17)	
C12	0.7267 (8)	0.4976 (2)	0.2513 (5)	0.0748 (19)	
H12	0.7319	0.5329	0.2311	0.090*	
C13	0.6920 (8)	0.4558 (2)	0.1925 (4)	0.0687 (17)	
H13	0.6745	0.4625	0.1322	0.082*	
C14	0.6833 (6)	0.4036 (2)	0.2238 (4)	0.0486 (13)	
N4	0.1005 (5)	0.30363 (16)	0.0850 (3)	0.0481 (10)	
C15	0.2355 (7)	0.3308 (2)	0.0382 (4)	0.0585 (15)	
H15A	0.3473	0.3244	0.0721	0.070*	
H15B	0.2356	0.3136	−0.0185	0.070*	
C16	0.2155 (8)	0.3893 (2)	0.0237 (4)	0.080 (2)	
H16A	0.2158	0.4073	0.0799	0.096*	
H16B	0.1058	0.3964	−0.0116	0.096*	
C17	0.3571 (8)	0.4115 (3)	−0.0218 (5)	0.093 (2)	
H17A	0.3565	0.3932	−0.0777	0.111*	
H17B	0.4665	0.4040	0.0137	0.111*	
C18	0.3428 (11)	0.4709 (3)	−0.0380 (8)	0.179 (5)	
H18A	0.3498	0.4896	0.0173	0.268*	
H18B	0.2345	0.4788	−0.0727	0.268*	
H18C	0.4350	0.4826	−0.0689	0.268*	
C19	0.0817 (6)	0.3310 (2)	0.1697 (3)	0.0524 (14)	
H19A	−0.0017	0.3112	0.1984	0.063*	
H19B	0.0352	0.3668	0.1567	0.063*	
C20	0.2431 (7)	0.3364 (2)	0.2329 (4)	0.0604 (15)	
H20A	0.3298	0.3548	0.2044	0.073*	
H20B	0.2866	0.3008	0.2503	0.073*	
C21	0.2101 (9)	0.3676 (3)	0.3135 (4)	0.0762 (19)	
H21A	0.1071	0.3534	0.3338	0.091*	
H21B	0.3053	0.3615	0.3597	0.091*	
C22	0.1894 (11)	0.4244 (3)	0.3004 (6)	0.117 (3)	
H22A	0.2898	0.4389	0.2793	0.176*	
H22B	0.1744	0.4413	0.3551	0.176*	
H22C	0.0902	0.4311	0.2579	0.176*	
C23	0.1534 (6)	0.2459 (2)	0.0986 (4)	0.0578 (15)	
H23A	0.2673	0.2450	0.1322	0.069*	
H23B	0.1620	0.2300	0.0415	0.069*	
C24	0.0357 (7)	0.21084 (19)	0.1452 (5)	0.0713 (19)	
H24A	0.0326	0.2249	0.2040	0.086*	
H24B	−0.0800	0.2128	0.1138	0.086*	
C25	0.0915 (9)	0.1527 (3)	0.1516 (6)	0.100 (2)	
H25A	0.2143	0.1517	0.1711	0.120*	
H25B	0.0349	0.1355	0.1967	0.120*	
C26	0.0580 (11)	0.1222 (3)	0.0743 (6)	0.130 (3)	
H26A	0.1130	0.1388	0.0288	0.195*	
H26B	−0.0639	0.1207	0.0562	0.195*	
H26C	0.1020	0.0864	0.0850	0.195*	
C27	−0.0763 (6)	0.3079 (2)	0.0309 (3)	0.0546 (14)	
H27A	−0.1613	0.2942	0.0657	0.066*	
H27B	−0.1017	0.3457	0.0191	0.066*	
C28	−0.0946 (8)	0.2782 (3)	−0.0552 (4)	0.095 (2)	
H28A	0.0029	0.2864	−0.0857	0.114*	
H28B	−0.0947	0.2397	−0.0441	0.114*	
C29	−0.2574 (10)	0.2935 (3)	−0.1122 (5)	0.098 (2)	
H29A	−0.2581	0.3323	−0.1199	0.117*	
H29B	−0.2560	0.2773	−0.1697	0.117*	
C30	−0.4061 (10)	0.2790 (4)	−0.0820 (5)	0.132 (3)	
H30A	−0.4054	0.2921	−0.0231	0.198*	
H30B	−0.4159	0.2404	−0.0824	0.198*	
H30C	−0.5021	0.2942	−0.1194	0.198*	

### Atomic displacement parameters (Å^2^)

**Table:**

	*U*^11^	*U*^22^	*U*^33^	*U*^12^	*U*^13^	*U*^23^
S1	0.0514 (8)	0.0594 (7)	0.0391 (7)	0.0010 (8)	0.0035 (6)	0.0034 (7)
O1	0.078 (3)	0.066 (2)	0.042 (3)	−0.003 (2)	0.006 (2)	0.003 (2)
O2	0.220 (7)	0.058 (3)	0.109 (5)	−0.020 (3)	0.003 (4)	−0.002 (3)
O3	0.130 (5)	0.089 (3)	0.073 (4)	0.000 (3)	0.000 (3)	−0.023 (3)
N1	0.050 (3)	0.059 (3)	0.044 (3)	0.003 (2)	0.008 (2)	−0.001 (2)
N2	0.050 (3)	0.062 (3)	0.044 (3)	0.002 (2)	0.004 (2)	0.006 (2)
N3	0.095 (4)	0.071 (4)	0.079 (5)	−0.004 (3)	−0.001 (3)	−0.008 (4)
C1	0.045 (3)	0.065 (4)	0.063 (4)	−0.001 (3)	0.007 (3)	−0.001 (4)
C2	0.090 (5)	0.054 (4)	0.082 (5)	−0.015 (3)	−0.001 (4)	−0.008 (4)
C3	0.124 (7)	0.068 (5)	0.110 (8)	−0.012 (4)	0.001 (6)	0.006 (5)
C4	0.091 (6)	0.097 (6)	0.108 (7)	−0.029 (5)	0.010 (5)	−0.031 (6)
C5	0.094 (6)	0.096 (5)	0.076 (5)	−0.016 (4)	0.016 (4)	−0.030 (5)
C6	0.056 (4)	0.074 (4)	0.060 (4)	−0.004 (3)	0.004 (3)	−0.012 (4)
C7	0.034 (3)	0.066 (4)	0.057 (4)	0.002 (3)	0.012 (3)	−0.001 (3)
C8	0.033 (3)	0.069 (4)	0.041 (3)	0.006 (3)	0.009 (2)	0.005 (3)
C9	0.038 (3)	0.059 (3)	0.041 (4)	−0.003 (3)	0.004 (3)	0.001 (3)
C10	0.050 (3)	0.063 (4)	0.049 (4)	0.005 (3)	0.003 (3)	0.002 (3)
C11	0.071 (5)	0.052 (3)	0.064 (4)	0.000 (3)	0.005 (4)	−0.011 (4)
C12	0.091 (5)	0.062 (4)	0.069 (5)	0.007 (3)	0.001 (4)	0.014 (4)
C13	0.084 (5)	0.061 (4)	0.059 (4)	−0.003 (3)	0.001 (3)	0.010 (3)
C14	0.044 (3)	0.054 (3)	0.048 (4)	0.007 (3)	0.002 (3)	0.008 (3)
N4	0.035 (2)	0.068 (3)	0.041 (3)	0.004 (2)	0.0049 (19)	−0.007 (2)
C15	0.048 (3)	0.075 (4)	0.054 (4)	−0.003 (3)	0.011 (3)	−0.001 (3)
C16	0.061 (4)	0.100 (5)	0.081 (5)	0.008 (4)	0.017 (4)	0.031 (4)
C17	0.057 (4)	0.128 (6)	0.089 (6)	−0.023 (4)	−0.002 (4)	0.039 (5)
C18	0.107 (8)	0.141 (7)	0.284 (15)	−0.005 (6)	0.013 (8)	0.150 (9)
C19	0.044 (3)	0.071 (3)	0.043 (3)	0.001 (3)	0.007 (3)	−0.005 (3)
C20	0.049 (3)	0.073 (4)	0.058 (4)	−0.006 (3)	0.003 (3)	−0.003 (3)
C21	0.064 (4)	0.105 (5)	0.056 (5)	−0.006 (4)	−0.003 (3)	−0.023 (4)
C22	0.101 (6)	0.095 (5)	0.154 (10)	−0.012 (5)	0.011 (6)	−0.050 (6)
C23	0.038 (3)	0.057 (3)	0.076 (4)	0.005 (3)	0.000 (3)	−0.005 (3)
C24	0.051 (4)	0.058 (3)	0.103 (6)	−0.002 (3)	0.004 (4)	0.006 (4)
C25	0.079 (5)	0.090 (5)	0.127 (8)	0.000 (4)	−0.003 (5)	0.003 (5)
C26	0.134 (8)	0.134 (6)	0.111 (8)	0.058 (6)	−0.027 (6)	−0.051 (6)
C27	0.038 (3)	0.081 (4)	0.044 (3)	0.007 (3)	0.000 (2)	−0.006 (3)
C28	0.058 (4)	0.175 (7)	0.048 (4)	0.002 (4)	−0.008 (3)	−0.024 (4)
C29	0.094 (6)	0.125 (6)	0.067 (5)	−0.027 (5)	−0.014 (4)	−0.006 (5)
C30	0.069 (5)	0.238 (11)	0.084 (6)	0.004 (6)	−0.012 (4)	−0.033 (7)

### Geometric parameters (Å, °)

**Table:**

S1—C9	1.725 (5)	C17—H17A	0.9800
S1—C8	1.799 (5)	C17—H17B	0.9800
O1—C7	1.245 (6)	C18—H18A	0.9700
O2—N3	1.216 (7)	C18—H18B	0.9700
O3—N3	1.226 (7)	C18—H18C	0.9700
N1—C8	1.335 (6)	C19—C20	1.509 (7)
N1—C7	1.365 (7)	C19—H19A	0.9800
N2—C8	1.327 (6)	C19—H19B	0.9800
N2—C14	1.360 (7)	C20—C21	1.525 (8)
N3—C11	1.441 (7)	C20—H20A	0.9800
C1—C2	1.383 (8)	C20—H20B	0.9800
C1—C6	1.388 (8)	C21—C22	1.444 (8)
C1—C7	1.480 (7)	C21—H21A	0.9800
C2—C3	1.380 (8)	C21—H21B	0.9800
C2—H2	0.9400	C22—H22A	0.9700
C3—C4	1.394 (11)	C22—H22B	0.9700
C3—H3	0.9400	C22—H22C	0.9700
C4—C5	1.380 (10)	C23—C24	1.530 (7)
C4—H4	0.9400	C23—H23A	0.9800
C5—C6	1.361 (8)	C23—H23B	0.9800
C5—H5	0.9400	C24—C25	1.521 (8)
C6—H6	0.9400	C24—H24A	0.9800
C9—C10	1.390 (7)	C24—H24B	0.9800
C9—C14	1.402 (7)	C25—C26	1.415 (10)
C10—C11	1.381 (7)	C25—H25A	0.9800
C10—H10	0.9400	C25—H25B	0.9800
C11—C12	1.388 (8)	C26—H26A	0.9700
C12—C13	1.394 (8)	C26—H26B	0.9700
C12—H12	0.9400	C26—H26C	0.9700
C13—C14	1.398 (7)	C27—C28	1.517 (8)
C13—H13	0.9400	C27—H27A	0.9800
N4—C19	1.505 (6)	C27—H27B	0.9800
N4—C23	1.513 (6)	C28—C29	1.511 (9)
N4—C15	1.531 (6)	C28—H28A	0.9800
N4—C27	1.537 (6)	C28—H28B	0.9800
C15—C16	1.487 (7)	C29—C30	1.373 (9)
C15—H15A	0.9800	C29—H29A	0.9800
C15—H15B	0.9800	C29—H29B	0.9800
C16—C17	1.509 (8)	C30—H30A	0.9700
C16—H16A	0.9800	C30—H30B	0.9700
C16—H16B	0.9800	C30—H30C	0.9700
C17—C18	1.511 (9)		
			
C9—S1—C8	88.4 (3)	C17—C18—H18C	109.5
C8—N1—C7	118.3 (5)	H18A—C18—H18C	109.5
C8—N2—C14	110.8 (5)	H18B—C18—H18C	109.5
O2—N3—O3	121.4 (7)	N4—C19—C20	115.6 (4)
O2—N3—C11	119.7 (7)	N4—C19—H19A	108.4
O3—N3—C11	118.9 (6)	C20—C19—H19A	108.4
C2—C1—C6	117.5 (6)	N4—C19—H19B	108.4
C2—C1—C7	119.3 (6)	C20—C19—H19B	108.4
C6—C1—C7	123.2 (5)	H19A—C19—H19B	107.4
C3—C2—C1	121.7 (7)	C19—C20—C21	110.7 (5)
C3—C2—H2	119.1	C19—C20—H20A	109.5
C1—C2—H2	119.1	C21—C20—H20A	109.5
C2—C3—C4	119.0 (7)	C19—C20—H20B	109.5
C2—C3—H3	120.5	C21—C20—H20B	109.5
C4—C3—H3	120.5	H20A—C20—H20B	108.1
C5—C4—C3	120.0 (7)	C22—C21—C20	114.8 (6)
C5—C4—H4	120.0	C22—C21—H21A	108.6
C3—C4—H4	120.0	C20—C21—H21A	108.6
C6—C5—C4	119.6 (8)	C22—C21—H21B	108.6
C6—C5—H5	120.2	C20—C21—H21B	108.6
C4—C5—H5	120.2	H21A—C21—H21B	107.5
C5—C6—C1	122.1 (6)	C21—C22—H22A	109.5
C5—C6—H6	118.9	C21—C22—H22B	109.5
C1—C6—H6	118.9	H22A—C22—H22B	109.5
O1—C7—N1	125.4 (6)	C21—C22—H22C	109.5
O1—C7—C1	120.2 (6)	H22A—C22—H22C	109.5
N1—C7—C1	114.4 (5)	H22B—C22—H22C	109.5
N2—C8—N1	121.7 (5)	N4—C23—C24	116.1 (4)
N2—C8—S1	114.1 (4)	N4—C23—H23A	108.3
N1—C8—S1	124.2 (4)	C24—C23—H23A	108.3
C10—C9—C14	121.2 (5)	N4—C23—H23B	108.3
C10—C9—S1	128.6 (4)	C24—C23—H23B	108.3
C14—C9—S1	110.2 (4)	H23A—C23—H23B	107.4
C11—C10—C9	118.1 (5)	C25—C24—C23	113.0 (5)
C11—C10—H10	120.9	C25—C24—H24A	109.0
C9—C10—H10	120.9	C23—C24—H24A	109.0
C10—C11—C12	122.1 (6)	C25—C24—H24B	109.0
C10—C11—N3	119.4 (6)	C23—C24—H24B	109.0
C12—C11—N3	118.5 (6)	H24A—C24—H24B	107.8
C11—C12—C13	119.6 (6)	C26—C25—C24	116.2 (7)
C11—C12—H12	120.2	C26—C25—H25A	108.2
C13—C12—H12	120.2	C24—C25—H25A	108.2
C12—C13—C14	119.4 (6)	C26—C25—H25B	108.2
C12—C13—H13	120.3	C24—C25—H25B	108.2
C14—C13—H13	120.3	H25A—C25—H25B	107.4
N2—C14—C13	123.9 (5)	C25—C26—H26A	109.5
N2—C14—C9	116.5 (5)	C25—C26—H26B	109.5
C13—C14—C9	119.6 (6)	H26A—C26—H26B	109.5
C19—N4—C23	111.9 (4)	C25—C26—H26C	109.5
C19—N4—C15	111.5 (4)	H26A—C26—H26C	109.5
C23—N4—C15	107.1 (4)	H26B—C26—H26C	109.5
C19—N4—C27	104.7 (4)	C28—C27—N4	114.7 (4)
C23—N4—C27	111.0 (4)	C28—C27—H27A	108.6
C15—N4—C27	110.7 (4)	N4—C27—H27A	108.6
C16—C15—N4	116.3 (4)	C28—C27—H27B	108.6
C16—C15—H15A	108.2	N4—C27—H27B	108.6
N4—C15—H15A	108.2	H27A—C27—H27B	107.6
C16—C15—H15B	108.2	C29—C28—C27	111.3 (6)
N4—C15—H15B	108.2	C29—C28—H28A	109.4
H15A—C15—H15B	107.4	C27—C28—H28A	109.4
C15—C16—C17	111.3 (5)	C29—C28—H28B	109.4
C15—C16—H16A	109.4	C27—C28—H28B	109.4
C17—C16—H16A	109.4	H28A—C28—H28B	108.0
C15—C16—H16B	109.4	C30—C29—C28	116.0 (7)
C17—C16—H16B	109.4	C30—C29—H29A	108.3
H16A—C16—H16B	108.0	C28—C29—H29A	108.3
C16—C17—C18	113.4 (6)	C30—C29—H29B	108.3
C16—C17—H17A	108.9	C28—C29—H29B	108.3
C18—C17—H17A	108.9	H29A—C29—H29B	107.4
C16—C17—H17B	108.9	C29—C30—H30A	109.5
C18—C17—H17B	108.9	C29—C30—H30B	109.5
H17A—C17—H17B	107.7	H30A—C30—H30B	109.5
C17—C18—H18A	109.5	C29—C30—H30C	109.5
C17—C18—H18B	109.5	H30A—C30—H30C	109.5
H18A—C18—H18B	109.5	H30B—C30—H30C	109.5
			
C6—C1—C2—C3	−0.1 (9)	N3—C11—C12—C13	176.9 (6)
C7—C1—C2—C3	−179.9 (6)	C11—C12—C13—C14	0.5 (9)
C1—C2—C3—C4	2.4 (11)	C8—N2—C14—C13	177.6 (5)
C2—C3—C4—C5	−1.7 (12)	C8—N2—C14—C9	−0.6 (6)
C3—C4—C5—C6	−1.3 (11)	C12—C13—C14—N2	−179.4 (5)
C4—C5—C6—C1	3.7 (10)	C12—C13—C14—C9	−1.3 (8)
C2—C1—C6—C5	−3.0 (8)	C10—C9—C14—N2	179.9 (5)
C7—C1—C6—C5	176.9 (5)	S1—C9—C14—N2	1.2 (6)
C8—N1—C7—O1	−0.6 (7)	C10—C9—C14—C13	1.7 (8)
C8—N1—C7—C1	−179.3 (4)	S1—C9—C14—C13	−177.1 (4)
C2—C1—C7—O1	3.1 (8)	C19—N4—C15—C16	−53.1 (6)
C6—C1—C7—O1	−176.7 (5)	C23—N4—C15—C16	−175.8 (5)
C2—C1—C7—N1	−178.1 (5)	C27—N4—C15—C16	63.1 (6)
C6—C1—C7—N1	2.0 (7)	N4—C15—C16—C17	179.3 (6)
C14—N2—C8—N1	179.1 (4)	C15—C16—C17—C18	−179.9 (7)
C14—N2—C8—S1	−0.3 (5)	C23—N4—C19—C20	64.5 (5)
C7—N1—C8—N2	177.6 (4)	C15—N4—C19—C20	−55.4 (6)
C7—N1—C8—S1	−3.1 (6)	C27—N4—C19—C20	−175.2 (5)
C9—S1—C8—N2	0.8 (4)	N4—C19—C20—C21	176.5 (5)
C9—S1—C8—N1	−178.5 (4)	C19—C20—C21—C22	−74.8 (7)
C8—S1—C9—C10	−179.7 (5)	C19—N4—C23—C24	57.3 (6)
C8—S1—C9—C14	−1.0 (4)	C15—N4—C23—C24	179.7 (5)
C14—C9—C10—C11	−1.2 (8)	C27—N4—C23—C24	−59.3 (6)
S1—C9—C10—C11	177.3 (4)	N4—C23—C24—C25	176.6 (5)
C9—C10—C11—C12	0.4 (8)	C23—C24—C25—C26	−75.7 (8)
C9—C10—C11—N3	−176.5 (5)	C19—N4—C27—C28	−174.3 (5)
O2—N3—C11—C10	175.3 (6)	C23—N4—C27—C28	−53.4 (6)
O3—N3—C11—C10	−6.4 (9)	C15—N4—C27—C28	65.4 (6)
O2—N3—C11—C12	−1.7 (9)	N4—C27—C28—C29	−168.3 (5)
O3—N3—C11—C12	176.6 (6)	C27—C28—C29—C30	−65.9 (9)
C10—C11—C12—C13	−0.1 (9)		

### Hydrogen-bond geometry (Å, °)

**Table:**

*D*—H···*A*	*D*—H	H···*A*	*D*···*A*	*D*—H···*A*
C28—H28A···O1^i^	0.98	2.24	3.166 (8)	157
C29—H29A···O2^ii^	0.98	2.39	3.353 (9)	166

Symmetry codes: (i) *x*−1/2, −*y*+1/2, *z*−1/2; (ii) *x*−1, −*y*+1, *z*−1/2.
